# Semiconductor to metal transition in two-dimensional gold and its van der Waals heterostack with graphene

**DOI:** 10.1038/s41467-020-15683-1

**Published:** 2020-05-06

**Authors:** Stiven Forti, Stefan Link, Alexander Stöhr, Yuran Niu, Alexei A. Zakharov, Camilla Coletti, Ulrich Starke

**Affiliations:** 1 Center for Nanotechnology Innovation @ NEST, Istituto Italiano di Tecnologia, Piazza San Silvestro 12, 56127 Pisa, Italy; 2 Max-Planck-Institut für Festkörperforschung, Heisenbergstr. 1, D-70569 Stuttgart, Germany; 3 MAX IV Laboratory, Lund University, P.O. Box 118, Lund, S-22100 Sweden; 4 Graphene Labs, Istituto Italiano di Tecnologia, via Morego 30, 16163 Genova, Italy

**Keywords:** Physics, Physics, Electronic structure of atoms and molecules, Electronic structure of atoms and molecules, Condensed-matter physics

## Abstract

The synthesis of two-dimensional (2D) transition metals has attracted growing attention for both fundamental and application-oriented investigations, such as 2D magnetism, nanoplasmonics and non-linear optics. However, the large-area synthesis of this class of materials in a single-layer form poses non-trivial difficulties. Here we present the synthesis of a large-area 2D gold layer, stabilized in between silicon carbide and monolayer graphene. We show that the 2D-Au ML is a semiconductor with the valence band maximum 50 meV below the Fermi level. The graphene and gold layers are largely non-interacting, thereby defining a class of van der Waals heterostructure. The 2D-Au bands, exhibit a 225 meV spin-orbit splitting along the $\overline {{\mathrm{\Gamma }}{\mathrm{K}}}$ direction, making it appealing for spin-related applications. By tuning the amount of gold at the SiC/graphene interface, we induce a semiconductor to metal transition in the 2D-Au, which has not yet been observed and hosts great interest for fundamental physics.

## Abstract (web summary)

Dimensionality of a material is a critical parameter to control its electronic properties. Here, the authors report that 2D gold transforms from a semiconductor, with valence band maximum 50 meV below the Fermi level, into a metal by tuning the number of layers from 1 to 2 in between graphene and SiC.

## Introduction

Lowering the dimensionality of a given compound has become a big hype in the field of material research, as this opens the possibility to explore and exploit properties, which are inaccessible in a bulk crystal. While several compounds can be relatively easily rendered two-dimensional (2D) with physical approaches^1–13^, many others are difficult to isolate. In particular, there are essentially no single-element materials other than graphite that can be easily exfoliated^14^. 2D metals see a vast field for applications, ranging from catalysis to sensing^15,16^, passing through enhanced magnetism^17–20^. This enticing applicative potential has prompted the development of various approaches for the synthesis of low-dimensional metals^15^. Several chemistry-based strategies have been implemented, however mostly yielding nanostructures of different shapes and geometries^15^. Physical methods have been shown to effectively yield monolayers of transition metals, typically on other metal surfaces^21–23^. The main focus of those types of investigations was the interactions between heavy atoms yielding large spin-orbit splitting of surface states, which might be useful for spintronic devices^24^. However, the strong interaction between the metallic substrate and the 2D layer typically hinders the possibility to access the intrinsic properties of the latter. Also, keeping the surface energy low enough to avoid the growth of multilayer islands is a non-trivial problem to address and often a capping medium solution must be adopted^25^.

In this article, we shed a light onto the electronic properties of two-dimensional gold (2D-Au), by synthesizing it via the intercalation of Au atoms at the heterointerface between the Si-terminated SiC(0001) surface and its C-rich (6$\sqrt 3$ × 6$\sqrt 3$)R30° reconstruction. Au atoms are arranged in a highly crystalline fashion and develop their own band dispersion, which is intrinsically two-dimensional. When a single layer of Au atoms is intercalated, a semiconductor (SC) crystal of hexagonal symmetry with the valence band maximum in ${\overline{\mathrm{K}}}_{{\mathrm{Au}}}$ close to the Fermi level and a saddle point in ${\overline{\mathrm{M}}}_{{\mathrm{Au}}}$ at about 400 meV is formed. These 2D states also exhibit a significant spin-orbit splitting, amounting to 225 meV, along the ${\overline{\Gamma {\mathrm{K}}}}$ dispersion direction. We show that the 2D semiconducting Au undergoes a transition to metal (M) when the number of intercalated Au layers goes from one to two. Transition metal atoms have already been intercalated underneath the buffer layer graphene^26–28^ or zerolayer graphene (ZLG) on SiC(0001) as well as under epitaxial graphene on Ni(111)^29^. The effects reported were doping of the graphene *π*-bands^30,31^ or mini-gaps opening in the graphene Dirac cone due to moiré superperiodicity^32^. It was recently shown that the interaction between the 5*d* orbitals of gold and the valence band of graphene can open a spin-orbit gap deep in the valence band of gold-intercalated graphene on SiC(0001)^33^. However, no evidence of two-dimensional dispersing bands stemming from the metal layer was ever reported, which would be indicative of a well-defined order of the film.

## Results

### Properties of the 2D-Au semiconducting phase

**Fig. 1:**
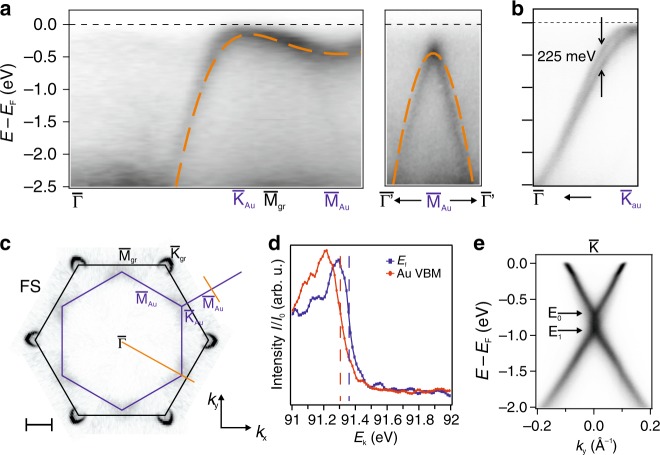
Electronic properties of 2D Gold. **a** ARPES cut along the diagonal orange line in panel **c**, showing only the Au bands dispersion. In the right panel the spectrum cuts along the short orange line in panel **c**. The orange dashed lines superimposed to the data are the TB bands as described in the text. **b** Au bands dispersion along the *k*_*y*_ direction highlighting their spin-orbit splitting Δ*SO*. The photon energy for panels **a** and **b** is 95 eV. **c** Fermi surface of the system measured by ARPES. The figure is the result of the sixfold symmetrisation of a measurement. Scale-bar corresponding to 0.5 Å^−1^. **d** Normalized line profiles extracted along the graphene *π*-band and the $\overline{\mathrm{K}}_{{\mathrm{Au}}}$ point (cf. Supplementary Fig. 1). **e** Dispersion measured along *k*_*y*_ at the $\overline{\mathrm{K}}_{{\mathrm{Gr}}}$ point with 40 eV photons, showing graphene’s Dirac cone.

Figure 1a, b displays the band structure of the SC 2D-Au, measured by angle-resolved photoemission spectroscopy (ARPES). Two spectral cuts are shown in Fig. 1a, which give the dispersion along the high symmetry directions $${\overline{\Gamma {\mathrm{K}}}} _{\mathrm{Au}}$$$${\overline{\mathrm{M}}}_{{\mathrm{Au}}}$$ and $${\overline{\Gamma}}^{\prime}$$$${\overline{{\mathrm{M}}}}_{{\mathrm{Au}}}$$$${\overline{\Gamma}}^{\prime}$$. From $${\overline{\Gamma }}$$ towards $${\overline{\mathrm{K}}}_{{\mathrm{Au}}}$$ the dispersion has a band velocity of (1.00 ± 0.05)⋅10^6^ ms^−1^ at a binding energy −1 eV for both the $${\overline{\Gamma {\mathrm{K}}}}$$ and $${\overline{\Gamma {\mathrm{M}}}}$$ directions, i.e., comparable with graphene’s band velocity in the vicinity of the Fermi level. From $$\overline{\mathrm{K}}_{{\mathrm{Au}}}$$ towards $$\overline{\mathrm{M}}_{{\mathrm{Au}}}$$, the band is instead rather flat. At $$\overline{\mathrm{K}}_{{\mathrm{Au}}}$$ we identify the maximum of the electronic dispersion. In order to locate it with precision, we perform the sigmoidal fit of the energy dispersion curves cutting through the apical part of the graphene *π*-bands and the $${\overline{\mathrm{K}}}_{{\mathrm{Au}}}$$ point. Such a fit with the respective energy locations is shown in Fig. 1d and it tells us that the 2D gold valence band maximum (VBM) is located at −50 meV. By performing a statistical analysis on the position of the VBM of 2D-Au for several samples, we find that the overall mean value falls just short of −70 meV (cf. Supplementary Note 1). The saddle point in $${\overline{\mathrm{M}}}_{{\mathrm{Au}}}$$ is found at −400 meV, meaning that the van Hove singularity (vHS) in the density of states is at an energy at reach for electronic measurements. No distinct crossing of the Fermi level is observed, in line with the missing Fermi surface contour in Fig. 1c. We observe a faint triangularly shaped spectral weight at the Fermi level, due to the intrinsic width of the band. By connecting the vertexes of the aforementioned intensities, we define the Brillouin zone (BZ) of the SC 2D-Au, drawn as a blue hexagon in Fig. 1c.

Photon energy dependent ARPES measurements were performed to probe a possible crystallographic periodicity along the ${\hat{z}}$ axis (see Supplementary Note 2), and confirmed the two-dimensional nature of the gold layer. Furthermore, a high-resolution close-up of the bands along the ${\overline{\Gamma {\mathrm{K}}}} _{\mathrm{Au}}$ direction shows that the states exhibit a spin-splitting which reaches the maximum value of 225 meV at −0.5 eV. It should be mentioned that since the states observed in this energy range and in this *k*-space portion have no correspondence in any other graphene/SiC(0001) based system^26,32,34^ they can solely be attributed to the interfacial Au layer.

The gold atoms at the interface affect the electronic properties of graphene^31^. A single gold layer acts as an electron donor to graphene, shifting the *π*-bands down until the Dirac point *E*_0_ is observed about 700 meV under the Fermi level, as visible from Fig. 1e, where we show an ARPES cut through graphene’s ${\overline{\mathrm{K}}}_{{\mathrm{Gr}}}$ point, taken perpendicular to the ${\overline{\Gamma {\mathrm{K}}}} _{\mathrm{Gr}}$ direction. In addition, the overall dielectric constant of the system is such that many-particle effects emerge and can be detected (cf. Supplementary Figs. 3 and 4). In particular, we extract an effective dielectric constant of *ε*_*eff*_ = 7 ± 1, which allow us to detect the plasmaron band^35^. The crossing energy of the plasmaron band is indicated as *E*_1_ and further details can be found in the Supplementary Note 3.

The size and shape of the 2D-Au BZ suggest that the Au atoms are arranged on a triangular lattice with the lattice parameter matching that of SiC(0001), i.e., 3.08 Å. As a further support to this hypothesis, we have observed with ARPES, graphene replica bands shifted by a SiC(0001) reciprocal lattice vector (cf. Supplementary Note 4).

We compared the observed bands to a tight binding (TB) model calculated on this 2D triangular lattice in the next-nearest neighbor (NNN) approximation:

1 $$g(\vec k) =	 \, - \!\varepsilon _0 - \gamma _{01}u_1(\vec k) - \gamma _{02}u_2(\vec k)\\ u_1(\vec k) =	 \,\, 2{\mathrm{cos}}(k_xa) + 4{\mathrm{cos}}(k_xa/2){\mathrm{cos}}(k_y\sqrt 3 a/2)\\ u_2(\vec k) =	 \,\, 2{\mathrm{cos}}(k_y\sqrt 3 a) + 4{\mathrm{cos}}(k_y\sqrt 3 a/2){\mathrm{cos}}(k_x3a/2)$$

where *a* is the lattice parameter, *γ*_01_ and *γ*_02_ are the NN and NNN hopping parameters, the first being of the order of 1 eV and *γ*_02_≃*γ*_01_/10. For the plot of Fig. 1a, b we set *γ*_01_ = 1.1 eV and *a* = 3.08 Å, that is the SiC(0001) lattice parameter.

Apparently, even this simple model describes the measured band dispersion quite well. In particular, the extremes are placed at the same *k*, confirming the right choice of *a*. The validity of the model also confirms the 2D nature of the states. However, some small deviations are present, especially around ${\overline {\mathrm{M}}}_{{\mathrm{Au}}}$.

Density functional theory (DFT) calculations have been published for this system by Chuang et al.^36^. These simulations show the formation of Au-interface related bands, similar to the ones observed in our experiment. There, it was presumed, that the structure of the interfacial Au is arranged such that every topmost Si atom of the SiC is bound to one Au atom, i.e. a (1 × 1) with respect to the SiC(0001). Similar to our experiment, their bands have the maximum in close proximity to the Fermi level, which indicates a semiconducting behavior.

Moreover, in ref. ^37^ the authors find a strong spin-splitting stemming from spin-orbit coupling. The spin texture there resembles a Rashba-type with a Rashba parameter of 1.638 eVÅ^−1^. In fact, we do find a strong correspondence to this in the experimental band structure (see Fig. 1b). Extracting the Rashba parameter from the experiments, we find a value of 2.17 eVÅ^−1^, which is in rather good agreement with theory.

**Fig. 2:**
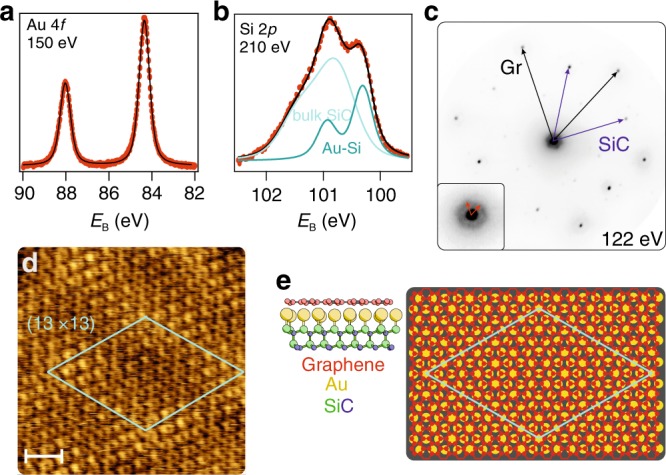
Structural properties of the interface. Core level spectra acquired on a Au-intercalated monolayer of epitaxial graphene on SiC(0001) of **a** Au 4*f* fitted with a single Voigt doublet, **b** Si 2*p* fitted with two Voigt doublets. The photon energies are indicated in the panels. **c** *μ*LEED pattern recorded at 122 eV on Au-intercalated graphene. The inset highlights the (13 × 13) pattern observed around the (00) spot. **d** STM topography image over a (5.2 × 5.2) nm^2^ region. The (13 × 13) moiré unit cell is indicated as a cyan diamond. Image recorded at *U*_sample_ = 100 mV and *I*_tunnel_ = 85 pA. Scale-bar 1 nm. **e** Ball-and-stick model of the gold atoms intercalated between graphene and SiC(0001). Side view and, on the right side, top view showing the (13 × 13) supercell.

In order to gather further information about the actual structure of the interfacial gold layer, we carried out X-ray photoemission spectroscopy (XPS), low-energy electron diffraction (LEED) and scanning tunneling microscopy (STM) measurements. Representative spectra of the Au 4*f* and Si 2*p* core levels are shown in Fig. 2a, b, respectively.

The details about the fitting parameters are reported in Supplementary Table 1. The Au 4*f* doublet indicates the presence of atoms in a single chemical environment. The binding energy that we measure is ~350 meV higher than for metallic gold and this excludes the presence of a substantial amount of metallic gold either on the surface or underneath graphene. In contrast, it indicates that Au is bound to Si at the interface, as confirmed by the Si 2*p* spectrum, which shows a component of bulk SiC and a surface component of Au–Si. Symmetry considerations allow us to conclude that, since the bands we observe are of *s*-*p* character, the Au atoms must interact with the 2*p* Si orbitals by their 5*d* electrons.

It should be noted that this does not represent an alloyed silicide layer, which is also reported to have a different (higher) binding energy^38^. What we observe is rather a significant electronic overlap between the topmost Si atoms of the substrate and the intercalated gold layer. The interaction with the substrate stabilizes the gold layer and induces the interfacial order. Notably, Au atoms deposited onto SiC(0001) are observed not to order on the (1 × 1)^38,39^, which implies the need for the graphene on top to impose the observed ordering and electronic structure.

The *μ*-LEED pattern of Fig. 2c shows that after the intercalation of Au atoms, only the graphene and SiC (1 × 1) are visible. In addition, a hexagonal halo oriented like graphene and 1/13 of its dimension is discernible. Atomically resolved STM carried out on such a sample, further corroborates the atomic arrangement. The STM image in Fig. 2d shows a region where the (13 × 13) superpotential is well visible and the graphene lattice is atomically resolved (see also Supplementary Fig. 9).

From the data reported above, we find that every Au atom is bound to a Si atom at the interface (cf. Supplementary Fig. 7), meaning that the Au is ordered in a (1 × 1) registry with respect to the SiC, as it was found from ARPES. In Fig. 2e we therefore report a ball-and-stick model of the interface in side and top view, to better illustrate the mutual arrangement between gold and graphene.

### Properties of the 2D-Au metallic phase

By extending the gold deposition time, it is possible to intercalate a double layer of gold (see Methods for more details). Going from a single Au layer to a Au bilayer does not only switch the doping of graphene from *n* + to slightly *p*, but it also switches the character of the 2D-Au electronic properties. In fact, we observe a transition from SC to a M behavior.

**Fig. 3:**
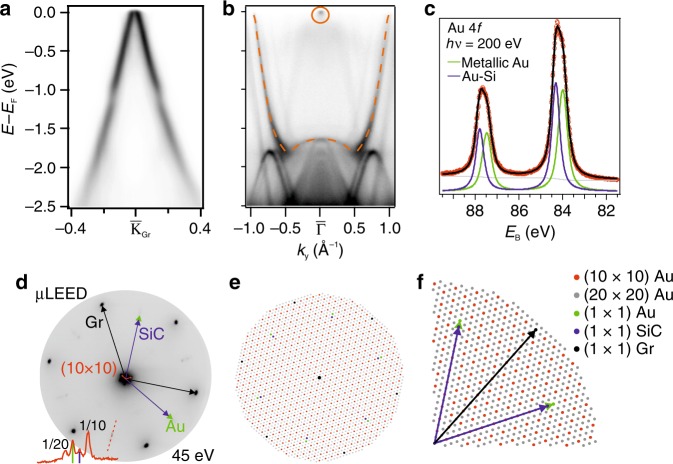
Properties of the *p*-phase Au-intercalated graphene. **a** ARPES spectrum of the graphene’s Dirac cone measured along *k*_*y*_, recorded with photons of 40 eV. **b** ARPES spectrum measured at 75 eV centered in ${\bar{\mathrm{\Gamma }}}$, showing the band structure of the intercalated gold along the *k*_*y*_ direction. **c** *μ*XPS spectrum of Au 4*f* doublet. **d** *μ*LEED pattern of the system. In the bottom-left corner the line profile along the red line is shown, highlighting the four diffraction spots visible and assigning them to SiC (blue), Au (green) and the 1/20 and 1/10 Au superperiodicities. **e** Sketch of the *μ*LEED with all the periodicities indicated. **f** Blow-up of one quadrant of panel **e**.

Figure 3 summarizes the electronic, chemical and structural properties of the M 2D-Au. In the first place, the graphene — shown in panel a — is marginally hole-doped with a density of states of (9 ± 1)⋅10^11^ cm^−2^, which, given a band velocity of (1.33 ± 0.05)⋅10^6^ ms^−1^, corresponds to a shift of the Fermi level by (−150 ± 10) meV with respect to the Dirac point. We as well observe the Rashba splitting at about −1 eV due to the interaction between the graphene and gold bands, as reported in ref. ^33^.

The interfacial gold still develops its own band dispersion, with a rich structure, as exemplary shown in Fig. 3b, where we show an ARPES spectrum recorded at 75 eV and centered in ${\overline{\Gamma }}$ along *k*_*y*_ direction, with reference to Fig. 1c. The downward parabolic states centered in ${\overline{\Gamma }}$ are gold quantum well states, as previously observed for very thin gold films on metal^24^. Similar states are as well observed for the SC phase, as visible from the ARPES data acquired at 75 and 40 eV, reported in Supplementary Figs. 2 and 5, respectively.

We mark the states reaching the Fermi level with the orange dashed line and a solid circle. The spectral weight at ${\overline{\Gamma }}$ at the Fermi level represents the bottom of a conduction band of the M 2D-Au and it is not related with the Shockley state of the Au(111) surface (cf. Supplementary Fig. 10). It was predicted in previously published calculations^36^, even if they considered three layers at the interface instead of two. Such a band structure implies that at any given small variation of the chemical potential, or applied external voltage, there will always be a finite electronic density of states, confirming the metallicity of this phase.

XPS measurements confirm the presence of a gold double layer, as does STM (see Supplementary Note 6). Figure 3c displays the Au 4*f* doublet fitted with two components. The ratio between the two is very close to one, indicating a symmetric distribution of the atoms between the first and the second layer.

Due to the favored interatomic forces, the intercalated gold bilayer shrinks its lattice parameter with respect to the monolayer. The gold atoms then arrange in a configuration closer to the bulk Au(111). This is illustrated by the *μ*-LEED data shown in Fig. 3d. A hexagonal pattern around the (00) spot is well visible. That vector is 1/10 of the Au reciprocal lattice vector. Yet, even a denser grid is visible, as the line profile (enlarged to be better seen) in the bottom-left part of the panel indicates.

Such a grid—represented in Fig. 3e, f—emerges from the Au/SiC(0001) interface and it corresponds to Au atoms being arranged as (20 × 20) over (19 × 19) SiC unit cells. The corresponding lattice parameter for the gold M-phase is extracted from several different LEED, *μ*LEED, ARPES, and STM measurements. The estimation of the lattice parameter is (2.93 ± 0.01) Å, about 1.6% larger than the nominal 2.883 Å of the Au(111).

**Fig. 4:**
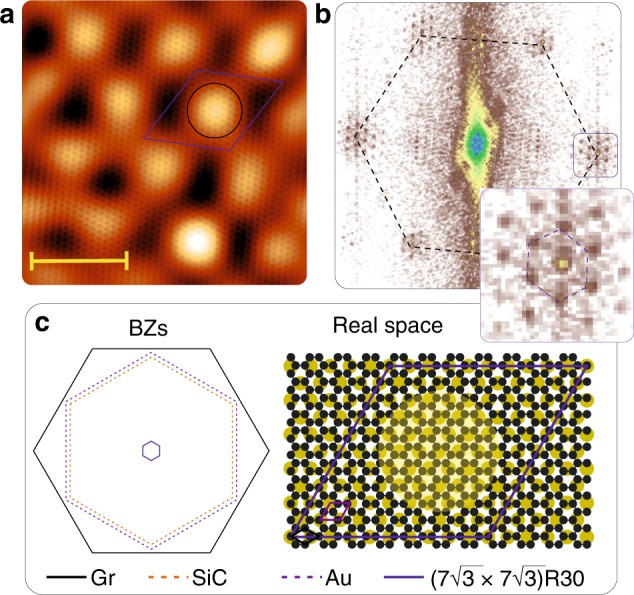
Moiré structure of M 2D-Au. **a** STM image 2D-FFT filtered *U*_sample_ = −105 mV *I*_tunnel_ = 325 pA. Scale-bar 3 nm. **b** 2D-FFT of the real-space image in panel **a**. In the inset: zoom-in with respect to the blue box in panel **b**. **c** Reciprocal and real-space arrangement between graphene and gold. All BZs are 90° rotated in order to match the alignment used for the ARPES description.

When such a gold bilayer interacts with graphene, it develops a superperiodicity half the size, which is observed in STM, as visible in the data reported in Fig. 4. The Gr/Au moiré is well identified in the topographical STM measurements of Fig. 4a. The graphene lattice is very well resolved as well and the superlattice unit cell is drawn in the image as a blue diamond. In Fig. 4b, the fast 2D Fourier transformation (2D-FFT) of the image (raw data in Supplementary Fig. 7) shows distinctively the graphene and superstructure periodicities and helps us conclude that (7$\sqrt 3$ × 7$\sqrt 3$)R30 graphene unit cells are arranged on (10 × 10) of gold, which is the strongest superperiodicity observed also in LEED (cf. Supplementary Fig. 8). The proposed model for the atomic arrangement of graphene and M 2D-Au is shown in Fig. 4c. The region within the supercell where the C-Au distance is larger is circled in panel **a** and indicated as a yellow disc in panel **c**. The difference in C-Au adsorption distance emerges from the local variation of the atomic registry between the lattices.

## Discussion

The intercalation method presented in this work yields a van der Waals heterostructure between a 2D semimetal, i.e., graphene and a 2D material formed by a triangular lattice of gold atoms, the electronic properties of which can be tuned, depending on the number of layers. By synthesizing a single layer of gold at the heterointerface between graphene and 6H-SiC(0001), we define a material with semiconductor character, exhibiting the maximum of the valence band at an average energy of 67 meV below the Fermi level. We have determined that the gold atoms arrange themselves in a highly ordered triangular lattice, mimicking the SiC(1 × 1) and develop their own band structure. Such an electronic band dispersion has a truly two-dimensional character and it is notably well reproduced by a simple TB model. Tuning the amount of intercalated gold from one to two layers, leads to a two-dimensional gold with metallic character. The interatomic forces within the gold layer make the lattice parameter shrink from 3.08 Å, to 2.93 Å, yet not quite relaxed into the Au(111) configuration, which has 2.88 Å as atomic spacing. Graphene is also affected by the underlying 2D gold in terms of electronic properties. Its doping level goes from *n* = (3.45 ± 0.05)⋅10^13^ cm^−2^ when on SC 2D-Au, to *p* = (9 ± 1)⋅10^11^ cm^−2^ when on M 2D-Au. The doping difference, i.e., a shift in the Dirac point position, is about 700 meV and is reflected almost entirely to a work function difference between the two phases (see Supplementary Fig. 6). The system therefore results in a double lateral junction: the graphene exhibit a *p*/*n*^+^ junction, in correspondence to the M/SC transition region of the 2D-Au, which represents a lateral Schottky contact. In addition, SC 2D-Au exhibits a vHS at about 400 meV from the Fermi edge, making this system a very appealing platform for the observation of collective phenomena at low temperatures. The whole structure is monolithic and does not require any transfer.

Besides the deep fundamental interest provided by this class of materials, we envision a vast portfolio of possible applications for this system, ranging from detection of THz radiation, to more sophisticated optoelectronic devices involving the spin degree of freedom. It will moreover serve as a platform for exploring non-linear optics effects in two-dimensional gold, a field so far essentially unexplored.

## Methods

### Sample preparation

Nominally on-axis oriented single crystalline single-side polished 6H-SiC *n*-type substrates were used in the present study and were purchased from SiCrystal GmbH. Polishing damages were removed by etching the substrate at 1500 °C in a purified H_2_ atmosphere. The SiC(0001) surfaces were graphitized by annealing the samples in argon atmosphere following the procedure introduced by Emtsev et al. in ref. ^40^. The ZLG develops upon annealing in Ar atmosphere at about 1400 °C for 10 min^41–45^. The formation of the ZLG results in a carbon-rich reconstruction with periodicity (6$\sqrt 3 \, \times \, 6\sqrt 3$)R30° with respect to the SiC(0001) unit cell^26–28^^[,46^. The samples were prepared by evaporating atomic Au from a Knudsen cell onto the ZLG surface, keeping the sample at temperatures between 600 and 700 °C and by subsequent annealing of the sample at temperatures between 800 and 850 °C. Keeping the sample at that temperature should favor the intercalation process over the diffusion of Au atoms over the surface and the consequent formation of clusters. Au is known to have a very high mobility on graphene^47^ so that deposition at room temperature followed by annealing leads to cluster formation^31^. Preparing the samples in the way described here helps in keeping the surface more clean. The single-layer gold is prepared in the aforementioned way by depositing a nominal amount of about 20 gold monolayers at a rate of 5 Å per minute. In this way the deposition takes about 10 min. For the gold bilayer a deposition time ranging from 20 to 25 min is used, employing the same rate. We point out that the intercalated samples are very stable in air (*τ* ≫ months) and that they do not require any particular protection from the atmospheric environment. In UHV conditions, they can be heated up to 850 °C before the intercalated gold starts to be deintercalated and desorbed from the surface.

### Measurements

The band structure of the system was mapped by means of ARPES at the end-station 1^2^ of the BESSY II (Berlin, Germany) synchrotron facility at a temperature of 90 K. The hemispherical electron analyzer used for the data harvesting was a Scienta R8000 with a diameter of 200 mm and an ultimate energy resolution of 1 meV. In our experiments the instrumental resolution was set to about 2.5 meV, hence smaller than the thermal broadening of the bands, i.e., about 7.5 meV. Additional ARPES measurements have been carried out at the I4 end-station of the MAXlab (Lund, Sweden) synchrotron facility with a SPECS Phoibos 100 analyzer. The aforementioned lines have also been used to acquire X-ray photoelectron spectroscopy (XPS) data. Preliminary studies to clarify the preparation procedures and to assess the sample’s quality were carried out in the home-laboratory at the MPI Stuttgart using a Specs Phoibos 150 analyzer and monochromatized He II radiation for the photoexcitation. Low-energy electron microscopy (LEEM) measurements, as well as micro-ARPES (*μ*ARPES), micro-LEED (*μ*LEED), and micro-XPS, were acquired at the beamline I311 beamline of the Maxlab using an Elmitec III microscope. More details about the performances of the microscope can be found elsewhere^28,48^. The scanning tunneling microscopy (STM) measurements were performed with an Omicron LT-STM microscope at the CNI@NEST in Pisa at a temperature of 78 K.

## Supplementary information

Supplementary Information

## Data Availability

The authors declare that the main data supporting the findings of this study are available within the article and its Supplementary Information files. Extra data are available from the corresponding author upon request.
