# Supplementary material for: Semiconductor to metal transition in two-dimensional gold and its van der Waals heterostack with graphene
Source: Nat Commun. 2020 May 6;11:2236. doi: 10.1038/s41467-020-15683-1 (PMC7203110; doi:10.1038/s41467-020-15683-1)
Supplement: Supplementary file 1 — Supplementary Information [file 41467_2020_15683_MOESM1_ESM.pdf]

Supplementary Information

Semiconductor to metal transition in two-dimensional gold and its van der Waals heterostack with graphene

S. Forti *et al.*

Semiconductor to metal transition in two-dimensional gold and its van der Waals heterostack with graphene

S. Forti[1,2,*], S. Link[2], A. Stöhr[2], Y. R. Niu[3], A. A. Zakharov[3], C. Coletti[1,4,*] and U. Starke[2]

[1] Center for Nanotechnology Innovation @ NEST, Istituto Italiano di Tecnologia, Piazza San Silvestro 12, 56127 Pisa, Italy
[2] Max-Planck-Institut für Festkörperforschung, Heisenbergstr. 1, D-70569 Stuttgart
[3] MAXIV laboratory, Lund University, P.O. Box 118, Lund, S-22100, Sweden
[4] Graphene Labs, Istituto Italiano di Tecnologia, via Morego 30, 16163 Genova, Italy

*Electronic address: stiven.forti@iit.it
*Electronic address: camilla.coletti@iit.it

Supplementary Discussion

Supplementary Note 1: Statistical analysis of the 2D ML Au semiconducting character

In order to present a robust and statistically solid set of measurements for proving the actual semiconductor character of the single-layer 2D gold, we carried out a systematic analysis of all our ARPES measurements, done on several gold-intercalated graphene samples, at different photon energies and at different synchrotron facilities. Such an extensive analysis allows us to present strong and consistent evidence of the semiconducting behavior of the 2D gold layer. The result of such analysis is reported in Supplementary Figure 1. In panel **a**, we show an exemplary ARPES measurement cutting through both the K points of graphene and 2D gold (cf. green line in the inset). In panel **b**, we report the energy distribution curves (EDCs) through the gray dashed lines at graphene's π-bands and at the $\overline{\mathrm{K}}_{\mathrm{Au}}$ point. The two profiles, i.e. the Fermi edge and the position of the gold valence band maximum (VBM), are fitted with a sigmoidal curve and the fit result is plotted as two vertical dashed lines on the graph. The same procedure was applied for every sample measured and the overall result is reported in the lower part of the figure, where the shaded region represents the error associated with the extracted value. The x axis of panel **c** shows the names of

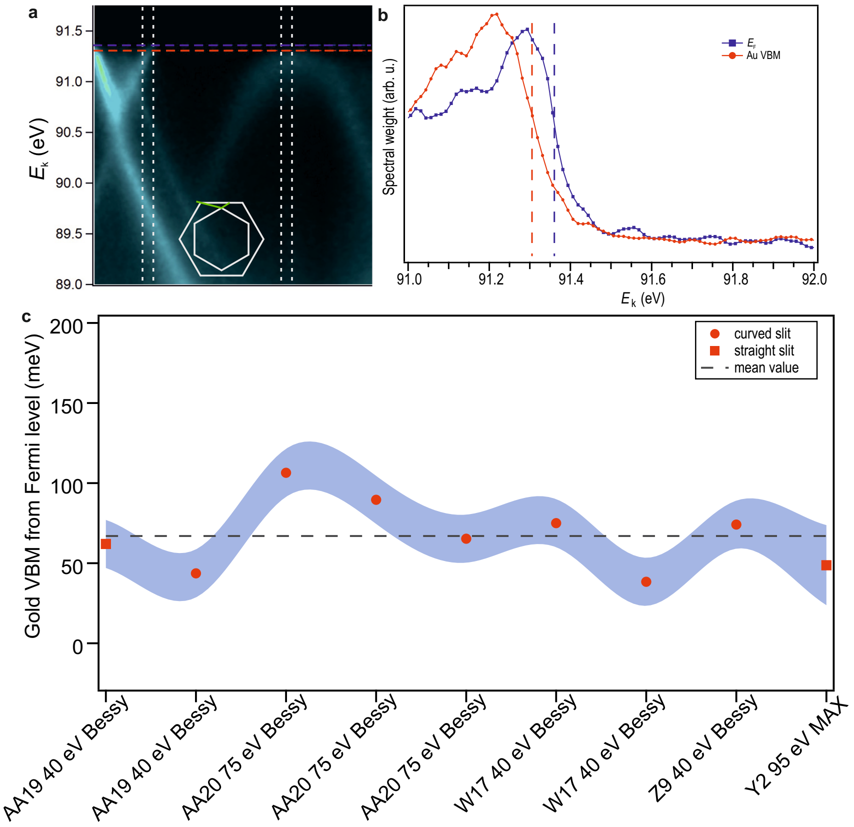

Supplementary Figure 1: **Analysis of the gold VBM energy position.** **a** ARPES cut along the green line in the inset, showing both the $\overline{\mathrm{K}}_{\mathrm{Gr}}$ and $\overline{\mathrm{K}}_{\mathrm{Au}}$ points. The horizontal lines account for the energy level as extracted by the sigmoidal fit of the profiles in panel **b**. **b** Profiles taken along the vertical grey-dashed lines in panel **a**. **c** distribution of the energy difference between the gold VBM and the Fermi level for different samples.

the samples measured, the photon energy and the facility at which they have been measured. The mean value extracted from this statistics is 67 meV, i.e. the energy distance from the 2D-Au VBM and the Fermi level.

Supplementary Note 2: Two-dimensionality of Au states

For completeness of what is reported in the main text, in Supplementary Figure 2**a** we show the TB bands for gold (graphene) calculated in the NNN approximation on a 2D triangular (honeycomb) lattice. The right side of panel **a** shows the density of states (DOS) derived from the TB model. The 2D-Au exhibits a Van Hove singularity at its $\overline{\mathrm{M}}$ point, which is located at about 400 meV from the Fermi level. This means that this system has an instability point which is at an energy reachable by electrical gating. In panel **b** we show the band dispersion of the system recorded with ARPES at 95 eV along the Au $\overline{\mathrm{MK\Gamma M}}$ direction. The energy scale is the same as for the theoretical bands in panel **a**. To prove the two-dimensionality of the gold bands, we show high-resolution ARPES data, collected with different photon energies to measure the dispersion of the k-vector along the z direction in reciprocal space. In panel **c** we show an exemplary of ARPES spectrum acquired along the Au $\overline{\mathrm{K\Gamma K'}}$ direction and centered in $\overline{\Gamma}$ with photon energy 75 eV. The spectral weight visible in the vicinity of $\overline{\Gamma}$ is due to gold replica bands as it will be further explained in sec. Supplementary Note 4:. The spectral weight at binding energy 1.7 eV, as traced by the dashed line in the panel, was extracted for every energy from 30 to 100 eV with energy step 5 eV and plotted as shown in panel **d**, following the relation that binds the k_z vector to the kinetik energy

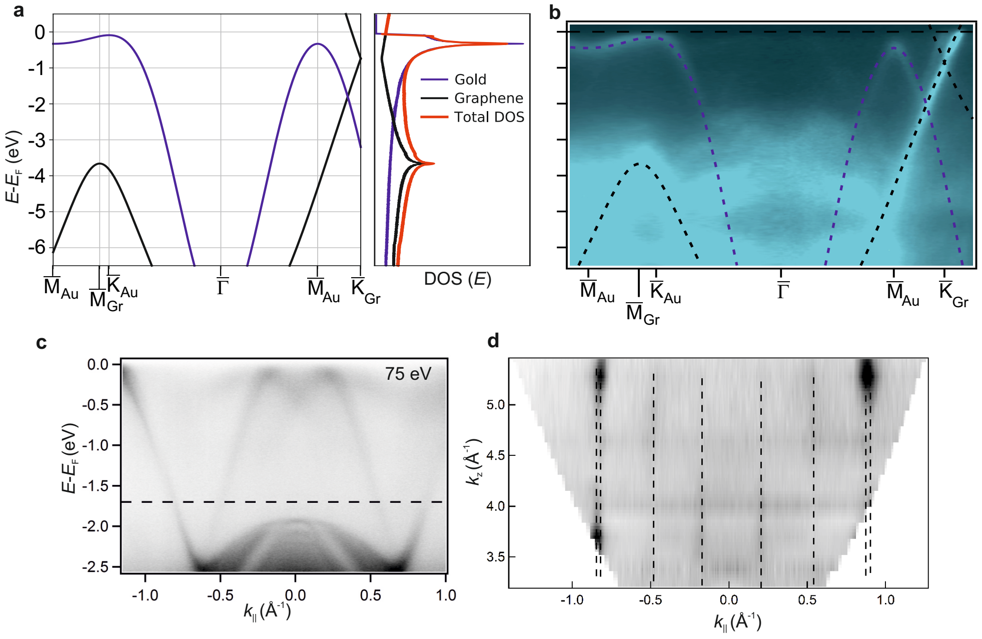

Supplementary Figure 2: **Overview of the Gr/2D-ML-Au band structure.** **a** NNN-TB bands of gold (blue) and graphene (black). In the right panel, the DOS of gold (blue), graphene (black) and the sum of the two (red) is shown in the same energy range. **b** ARPES spectrum acquired at 95 eV of the entire BZ of the Au-intercalated graphene. **c** ARPES spectrum acquired at 75 eV along the gold $\overline{\mathrm{K\Gamma K'}}$ direction. **d** k_z dispersion obtained by extracting the spectral weight of spectra similar to the one in panel **a** at 1.7 eV (see dashed line), recorded at photon energies from 30 to 100 eV with 5 eV of energy step.

and the emission angle: $k_z = \sqrt{\frac{2m}{\hbar}}\sqrt{E_{\rm kin}\cos^2(\theta) + V_0}$, where V_0 is the inner potential and θ the photoemission angle. We chose $V_0 = 17.5$ eV, according to Refs. [1,2] and considering the difference in the valence band minimum between gold and graphene as extracted from the TB calculations, i.e. about -1 eV. The absence of dispersion in the direction perpendicular to the surface of the k-vector indicates the actual two-dimensionality of the interfacial gold layer. The spectral weight visible towards the Fermi level in $\overline{\Gamma}$ is ascribed to gold replica bands, due to electron diffraction at the surface. The scattering vector corresponds to the reciprocal lattice vector of graphene, hence rotated by 30 degrees with respect to the gold BZ alignment (see also sec. Supplementary Note 4:).

Supplementary Note 3: The electronic properties of graphene over a single layer of gold

The effects of gold intercalation over the decoupled monolayer graphene on SiC(0001) have been already discussed in the past literature [3, 4]. Nevertheless, in this section we point out what are the effects of a very careful preparation on the properties of the intercalated graphene. The very careful preparation of the samples via the intercalation of precisely one Au monolayer with respect to the SiC(0001) atomic areal density, translates in a very clean ARPES signal and allows for the extraction of band parameters from the data. Supplementary Figure 3**a** shows the ARPES spectrum of the Dirac cone acquired along $\overline{\Gamma \mathrm{K}}_{\rm Gr}$ at 40 eV. The measured bands are accompained by the fitting of the positions of the maxima of the energy distribution curves, shown as green and red circles superimposed to the raw data. Two distinct dispersing states are fitted, as it is apparent from the figure. This effect has been reported for the first time in graphene by Bostwick and coworkers [5]. What is visible below the Dirac point is the energy dispersion of the plasmaron quasiparticle [6], i.e. a photohole that is coupled to a plasmon with similar group velocity. According to Ref. [4], by measuring the energy and momentum separation of the hole and plasmaron bands, one can estimate the effective dielectric constant of the investigated sample. We determined the energy and momentum spread of the diamond formed by the crossing of the hole and the plasmaron dispersions (cf. Supplementary Figure 4**a**). This is done in Supplementary Figure 3**b** and **c**, where the energy (**b**) and momentum (**c**) distribution curves of the Dirac cone are fitted with two Voigt functions, colored in gray. The two band traces are about 253 meV (**b**) and 0.02 Å^{-1} (**c**) apart from each other and the Dirac energy of the system is defined as E_0 and located (685±5) meV below the

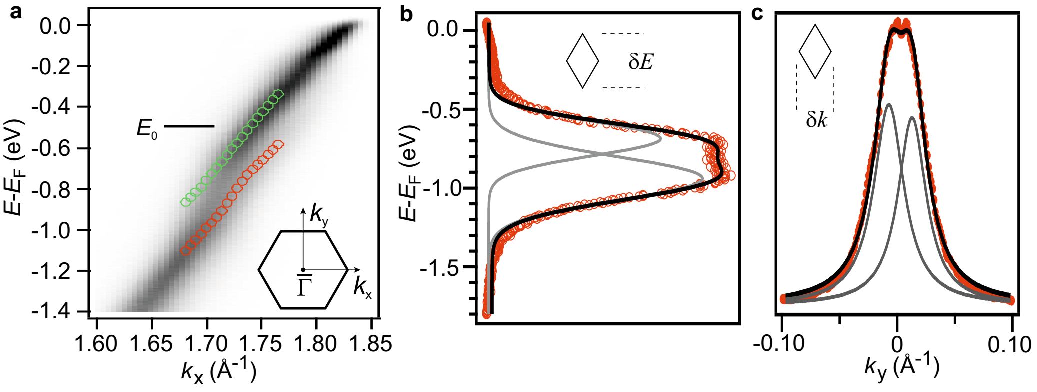

Supplementary Figure 3: **Observed plasmarons in graphene.** **a** ARPES spectrum at 40 eV of the Dirac cone along the k_x direction of a Au-intercalated epitaxial graphene, n-phase. The empty circles are fits of the peak position of the energy distribution curves. **b** and **c** line profiles of the plasmaron diamond, from which to extract the value of dielectric constant. See also Supplementary Figure 4.

Fermi level (cf. Supplementary Figure 4). To give an estimation of the effective coupling constant and effective dielectric constant, we compare our results with the work of Ref. [4]. However, we point out that the normalization of the diamond width in momentum space with respect to the Fermi momentum k_F is somewhat inconsistent since for such high doping level, the Dirac cone is strongly warped (see Supplementary Figure 4**b** and **c**). We therefore rely on the separation of the cones in energy. In this way, the α_{ee}, or graphene fine constant is found to be $\alpha_{ee} = 0.30 \pm 0.02$, which is translated into an effective dielectric constant $\epsilon_{eff} = 7 \pm 1$, meaning a substrate dielectric constant of $\epsilon_s = 2 * \epsilon_{eff} - 1 = 13 \pm 2$. Such a small effective dielectric constant enhances the e-e coupling and makes collective phenomena such as plasmarons observable. In their paper [4], Walter and coworkers reported a dielectric constant value about five times higher. This is simply because they started from a *p*-type Au intercalated graphene, which is induced by twice the amount of gold at the interface (cf. Fig. 3**c**), and counter *n*-doped it with K atoms in order to set the Fermi level of the system well above the Dirac point and thereby observe the plasmaron band. For completeness, we also extract the band parameters from the measured ARPES. To do this, we analyze the ARPES data of the graphene Dirac cone measured at 40 eV, as displayed in Supplementary Figure 4. In panel **a** we show the ARPES spectrum of the *n*-doped Dirac cone as in Fig. 1 of the main text, with the addition of dashed lines to distinguish between the hole (green) and plasmaron (red) dispersions. Panel **b** displays the Fermi surface, whereas panel **c** shows the amplitude of the Fermi vector as a function of the angle as centered in $\overline{\text{K}}$, highlighting the threefold symmetry of the Fermi surface and the dark corridor [7], where the intensity vanishes. The determination of the dielectric constant value by measuring the distance in *k*-space between the hole and plasmaron dispersion, normalized for the Fermi vector, is not a particularly precise way of doing it. Such a normalization procedure is indeed direction-dependent. Instead, the energy difference between the two dispersions is not, and

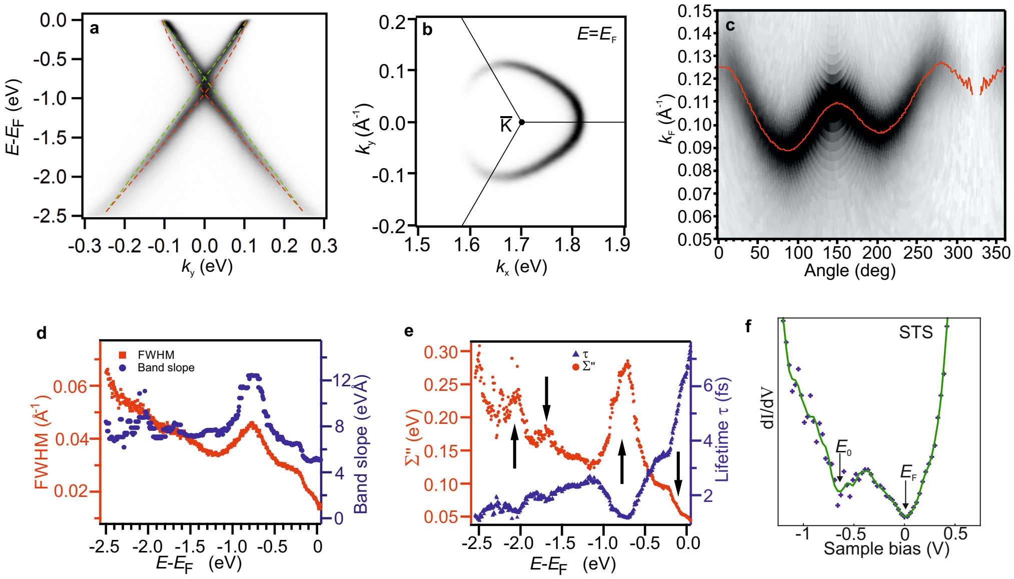

Supplementary Figure 4: **Band properties of the *n*-type graphene.** **a** ARPES spectrum of the graphene's Dirac cone measured perpendicular to the $\overline{\Gamma\text{K}}_{\text{Gr}}$ direction at 40 eV. Photohole and plasmaron dispersion are superimposed to guide the eye. **b** Fermi surface of graphene. **c** Angular dependence of the Fermi momentum. **d** FWHM and band slope of the Dirac cone extracted along the $\overline{\Gamma\text{K}}_{\text{Gr}}$ direction at 40 eV. **e** Imaginary part of the self energy and quasiparticle lifetime extracted from the FWHM (see text). **f** Scanning tunneling spectroscopy of Gr/SC Au/SiC(0001) acquired at 78 K. E_0 is the Dirac energy, as defined in the main text.

it is therefore more reliable. We extracted the band parameters by fitting the momentum dispersion curves (MDCs) with Lorentzian lineshapes. Supplementary Figure 4**d** shows the full width at half maximum (FWHM) of the bands and their slope $\partial E/\partial k$, in the energy range displayed in panel **a**. Panel **e** shows the imaginary component of the self energy and the photohole lifetime, which depend on the FWHM through the relation: $\Sigma'' = (\delta k/2)(\hbar v_{\mathrm{F}})$ and $\tau = 1/(v_{\mathrm{F}}\delta k)$, where δk is the FWHM and v_{F} the Fermi velocity. The self energy is a particularly sensitive quantity for measuring the interactions between quasiparticles. Indeed, a strong peak is visible in proximity of the Dirac point, corresponding to the photohole relaxation through the emission of a plasmon. The other two less pronounced peaks are ascribed to the interaction between graphene and gold bands, although in the dispersion no actual gap is observed. The first low-energy kink in the spectrum is due instead to quasiparticle relaxation via phonon emission. Supplementary Figure 4**f** displays the scanning tunneling spectroscopy (STS) spectrum of the semiconducting Au phase intercalated in between graphene and SiC(0001), acquired at 78 K. The Dirac point is observed at around -625 meV, in line with the ARPES measurements.

Supplementary Note 4: Interfacial atomic ordering derived from periodicity of ARPES replica bands

In this section we show the final state effects caused by the diffraction of photoemitted electrons as they exit the material.
In Supplementary Figure 5**a** a portion of the k-space is shown, recorded with ARPES at 40 eV centered in $\overline{\Gamma}$. At this binding energy, no intensity should be observable. However, some spectral weight clearly appears. Four Dirac cone profiles are distinguishable and correspond to graphene replica bands. Those replicas are induced by a reciprocal lattice vector with module and orientation corresponding to the SiC(0001) surface vector. They are therefore generated by the gold atoms arranged on the SiC (1×1), as shown in panel **c**. The intensity around $\overline{\Gamma}$ is due to gold replica bands. In this case the generator vector is the reciprocal lattice vector of graphene. In panel **b** we show an ARPES spectrum recorded at 75 eV along the gold $\overline{\mathrm{K}\Gamma\mathrm{K'}}$ direction with superimposed the theoretical gold SC bands derived from TB (in yellow) and the corresponding bands from the second BZ, displaced by a graphene reciprocal lattice vector (other colors). The matching is quite remarkable, confirming the proposed model and also justifying the residual intensity visible in the k_{z} vs. E plot in Supplementary Figure 2**c**.

Supplementary Note 5: Details about XPS data fitting

In this section we report the details about the fitting for the XPS data, as shown in Fig. 2 of the main text.
The Au 4f spectrum of the 2D SC phase can be well fitted with a single Voigt doublet lineshape (Fig. 2**a**). The Si 2p spectrum instead, needs a double Voigt doublet function (Fig. 2**b**). For the Au 4f, spin splitting was set to 3.68 eV and the branching ratio was 0.75. Standard constraints were set for the Si 2p fit, such as spin splitting of 0.63 eV and branching ratio of 0.5 for the single doublets. Within the respective doublets, both components were set to the same Lorentzian and Gaussian widths. The resulting fitting parameters can be found in Supplementary Table 1. The small Lorentzian and Gaussian widths for the Au 4f levels is indicative for the presence of only one chemical species of Au. The $4f_{7/2}$ component's binding energy of 84.35 eV is shifted by about 350 meV towards higher binding energy compared to metallic Au. This means firstly, that one can exclude the presence of Au clusters on the sample surface, like observed earlier [3]. Also, all Au atoms within the interface have the same chemical environment. Judging from the two very distinct chemical components in the Si 2p spectrum, it is reasonable to assume that these Au atoms are chemically bound to the topmost Si of the SiC(0001) surface. All components of the Si 2p spectrum show a Lorentzian broadening of 0.12 eV, which can be viewed standard for this core level. The

	E_B (eV)	ω_L (eV)	ω_G (eV)	ratio (%)
Au $4f_{7/2}$	84.35	0.15	0.20	
Si $2p_{3/2}$, bulk SiC	100.78	0.12	0.32	68
Si $2p_{3/2}$, Au-Si	100.31	0.12	0.13	32

Supplementary Table 1: Fit parameters of the Si $2p_{3/2}$ and the Au $4f_{7/2}$ components of the n-phase Au intercalated ZLG. The binding energies of the respective doublet partners are given by the spin splitting mentioned in the text, which was set as a constrain for the fit.

Gaussian width is 0.32 eV in the 100.78 eV binding energy doublet and 0.13 eV in the 100.31 eV doublet (both binding energies denote to the $2p_{3/2}$ components). A Gaussian broadening of 0.32 eV is generally observed for bulk SiC even if the experimental resolution is much better. However, the Gaussian width of Si $2p$ spectra can be much smaller in other materials, as it is the case for instance in the (7×7)-reconstruction of the Si(111) surface [8]. This peculiar broadness within the SiC system is not understood up until now, yet it can be seen as characteristic for bulk SiC. It is therefore reasonable to assign the narrower component to Si, which is bound to Au on the surface of the SiC. Au adsorbed on the (0001) surface of 4H-SiC have been investigated by Stoltz and coworkers [9]. They report a Si $2p$ component distribution, which is similar to our case, but with slightly different binding energies. There, they also assign the higher binding energy component to Si in the bulk of the SiC and the lower binding energy component to Si, which is bound to Au on the surface. The differences in binding energy can be caused by the different SiC polytype used here (6H-SiC) and by them (4H-SiC). Also the additional graphene layer on top in our case has influence on the band alignment and accompanied surface band bending in the SiC [10, 11], which

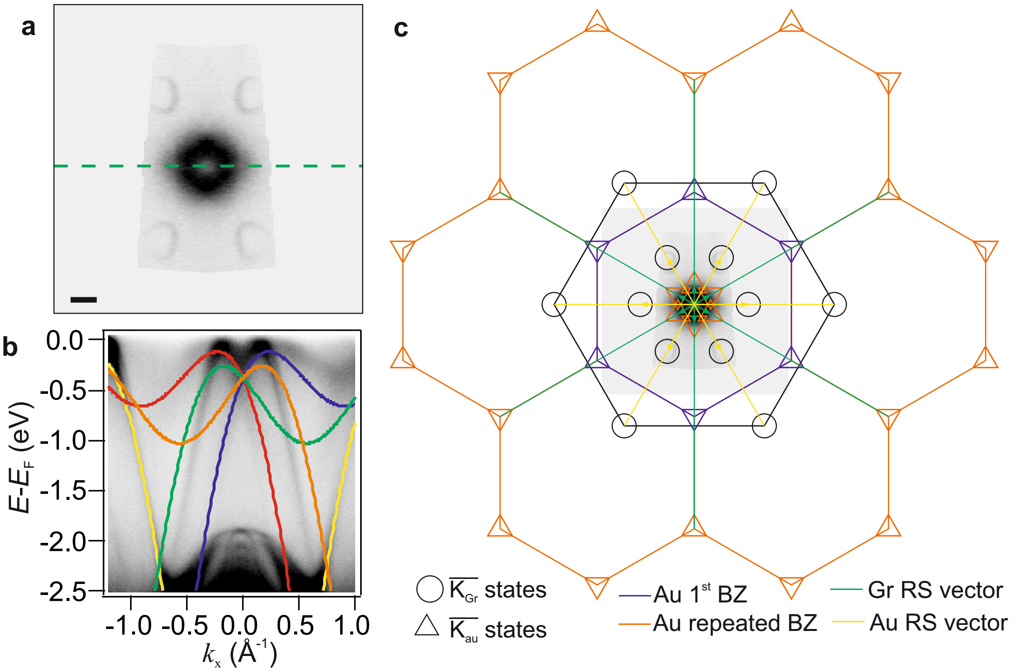

Supplementary Figure 5: **Gr/2D-ML-Au replica bands. a** ARPES constant energy surface close to the Fermi level measured about $\overline{\Gamma}$ at 40 eV. Scale-bar 0.1 Å^{-1}.**b** Model for the SC gold replica bands, having the graphene reciprocal lattice vector as generator. **c** Model for the graphene and gold replica bands, using SC-Au and graphene reciprocal space (RS) vectors as generators, respectively.

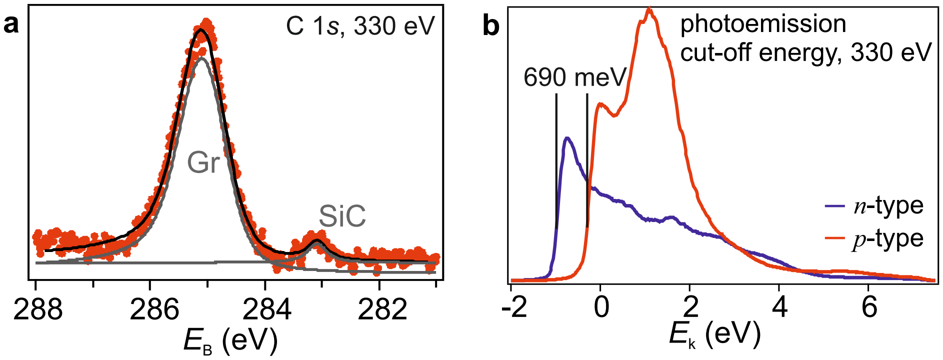

Supplementary Figure 6: **μXPS characterization of Gr/Au.** **a** μXPS spectrum of C 1s recorded on Gr/SC 2D Au/SiC(0001) at 330 eV. **b** Photoemission cut-off spectra recorded on Gr/Au/SiC(0001) regions where the graphene was n and p doped, respectively.

alters the observed binding energies.
It should be noted, that one can compare the intensity ratio of the signal produced by the two chemical Si species to other intercalation systems based on graphene on SiC(0001). The intensity ratio from Si in the bulk SiC and Si bound to Au is 68:32. In the case of H intercalation, the intensity ratio of Si in the bulk SiC to Si bound to H is 63:37 and 67:33 for photon energies 140 eV and 330 eV, respectively [12]. In this case, every Si atom on the surface of the SiC is bound to one H. Keeping in mind that the photon energy in the measurements presented here is 210 eV, it is reasonable to assume that also in the Au case here all Si atoms on the surface are bound to Au.
The decoupling of the graphene is well illustrated by the C 1s core level peak in Supplementary Figure 6**a**. The SiC peak binding energy is measured at about 283 eV, as observed in other intercalated systems [13–15]. The graphene peak is here fitted with a single Doniach-Ŝunjić (DS) centered at 285 eV with a gaussian width of 0.45 eV, which takes into account the different chemical environments present inside the supercell [15, 16]. The binding energy of the sp^2 carbon peak is increased by about 600 meV from the nominal peak position of 284.4 eV. Such a shift is mostly due to the n-type doping of about $n \sim 0.035$ electrons per graphene unit cell [17]. In turn, we observe this doping being accompanied by a decrease of work function. Being a 2D material, whenever the bands are rigidly moved up or down with respect to the Fermi level, a corresponding change in the work function must occur. This is actually observed and measured through the cut-off energy of the photoemitted electrons, displayed in Supplementary Figure 6**b**. We look at the low-kinetic energy part of the photoemission spectrum until we reach the edge where no electron is extracted from the material. In this way we can easily measure the difference in work function between regions which are n and p doped (corresponding to semiconducting and metallic gold phases, respectively). We measure an energy difference of (690±5) meV. Considering that the p-phase is almost neutral (cf. Fig. 3 in the main text), such a measure shows very well how tightly related the charge transfer and the variation of work function are in 2D materials.

Supplementary Note 6: STM measurements

In this section, we show scanning tunneling microscopy (STM) measurements of the gold-intercalated graphene in its SC and M phase. Supplementary Figure 7**a** shows a sample's region where the transition between the SC and M gold region is visible. The SC (M) region is shown zoomed-in in panel **b** (**e**), whereas the FFT-filtered and 2D-FFT of the zoomed-in region are shown in panel **c** and **d** (**f** and **g**), respectively. The superstructure on the M region is so large and strong that it can be seen even on the large-scale image. The corrugation of the M region pattern is indeed of

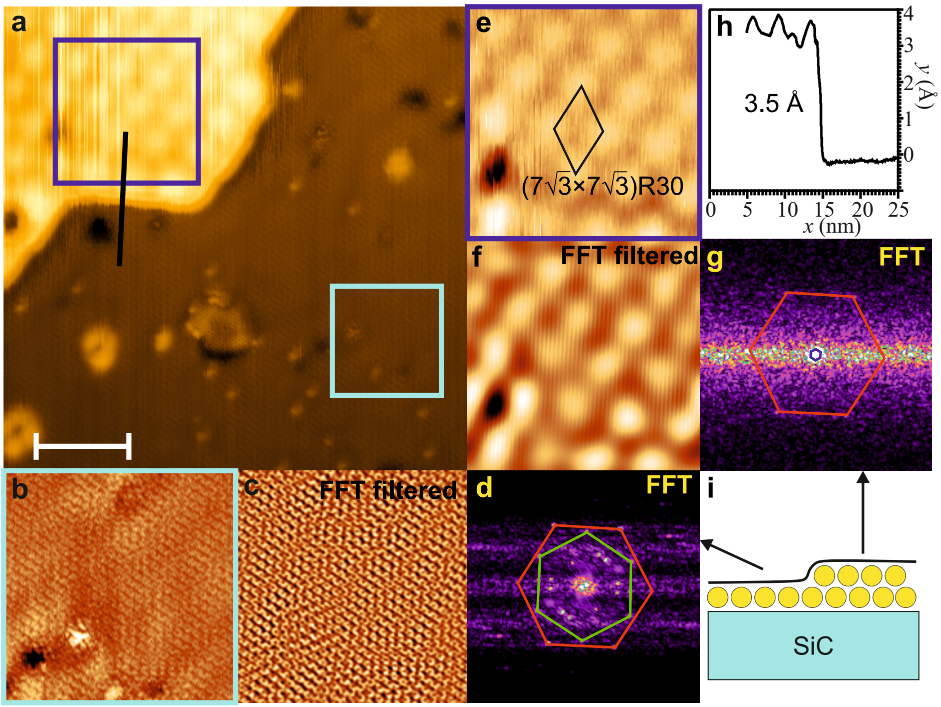

Supplementary Figure 7: **STM investigation of Gr/Au. a** STM topography of a SC/M transition region. Au-intercalated graphene recorded with 400 pA of constant tunnel current and -250 mV of tip voltage. Scale-bar 5 nm. Panels **b,c,d** and **e,f,g** show a zoomed-in portion of the scanned area corresponding to the frames on panel **a**, the FFT filtered image of the same region and the 2D-FFT of the image of the SC and M regions, respectively. Panel **h** shows the black line profile across the transition region between the SC and the M regions in panel **a**. **i** simple sketch of the system.

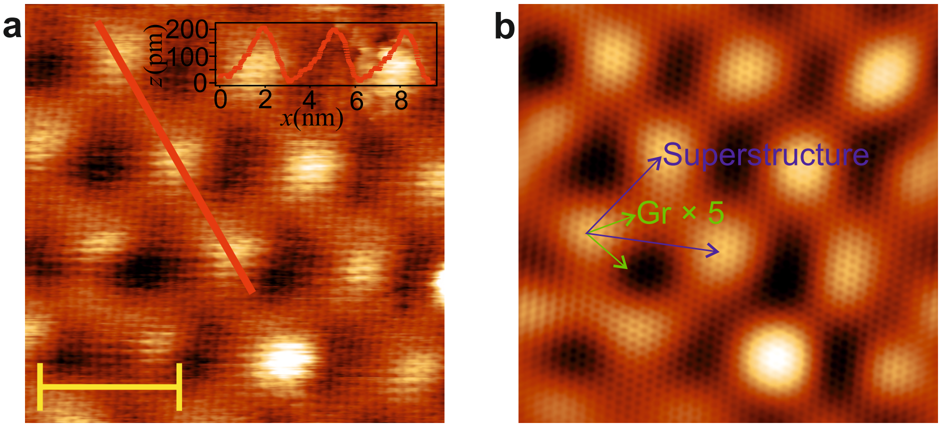

Supplementary Figure 8: **M-phase superstructure measure with STM. a** Raw topographical data. In the top-right inset, the line profile over the red line is shown. Scale-bar 3 nm. **b** 2D-FFT filtered image with indicated the lattice vectors of the superperiodicity and of graphene, the length of which has been increased by a factor 5.

the order of 200 pm, as visible also in the line profile of panel **h** (cf. also Supplementary Figure 8), while on the SC phase is about 25 pm. As visible from panel **e** and Supplementary Figure 8, the superstructure stemming from the M region has a periodicity of $(7\sqrt{3}\times7\sqrt{3})$R30 graphene unit cells over (10×10) gold, as also confirmed by μLEED measurements (see discussion in the main text). The height difference of about 3.5 Å observed between the two regions supports a model where only two gold layers are intercalated in the M phase.

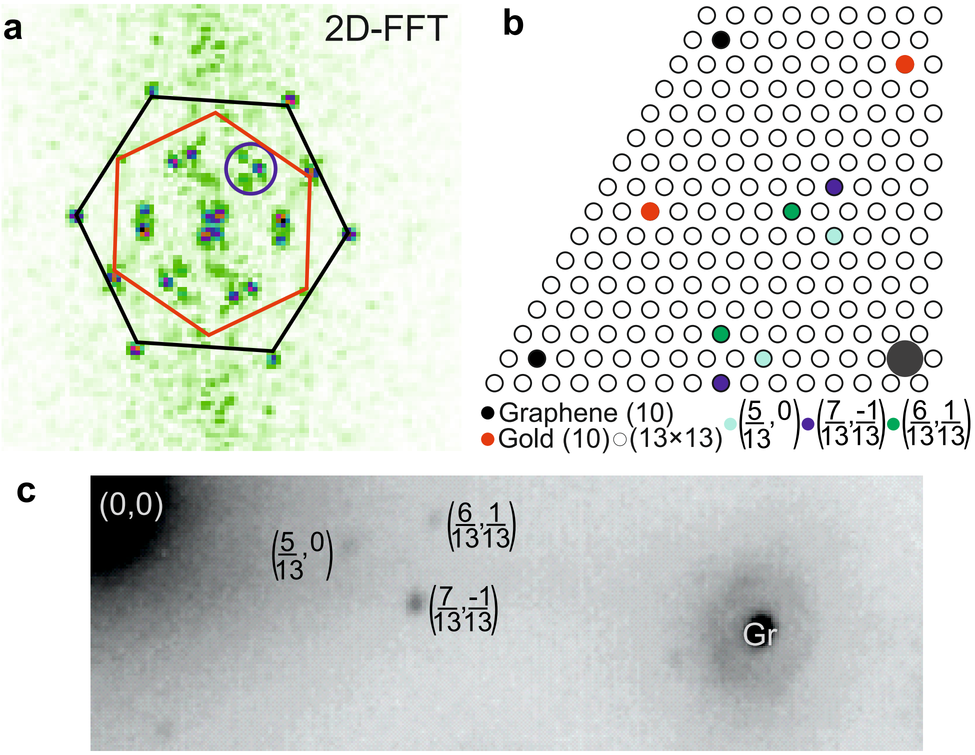

Supplementary Figure 9: **Comparison between the μLEED and STM results. a** 2D-FFT of SC Au phase, retrieved from Fig. 2**d**. Graphene and gold reciprocal lattice spots are indicated by a black and red hexagon, respectively. The spots stemming from the (13×13) periodicity are circled in blue. **b** Theoretical reciprocal lattice grid of the (13×13), representing the kinematic LEED pattern in the first quadrant. Highlighted are the spots visible in panel **a**. **c** portion of Fig.2**c** from the main text with enhanced contrast, to highlight the high order diffraction spots on the (13×13) grid.

The 2D fast Fourier transformed (FFT) pattern in Supplementary Figure 9**a** clearly shows the graphene and gold reciprocal lattice spots, indicated with a black and red hexagon, respectively. Other spots are also visible and they belong to the (13×13) grid, as explained in panel **b**, where the top-left quadrant of the theoretical LEED pattern up to the graphene's first diffraction order is shown. The blue circle in panel **a** encloses three spots, two of which are visible along every direction. The third and innermost spot is less visible in the FFT. As illustrated in panel **b**, those spots are the (7/13,-1/13), (6/13,1/13) and (5/13,0) reflexes of the (13×13) pattern. The same spots are measured as well by μLEED, as we prove by showing in panel **c** a portion of Fig.2**c** with enhanced contrast. We briefly point out that the (13×13) is a rather natural periodicity for the graphene on SiC system and it is often observed, also for other intercalated systems [15]. In Ref. [18] , for example, they have observed several different superperiodicities induced by the intercalated gold. Even the (13×13), but in that case, the gold was aligned with the graphene and it had a different

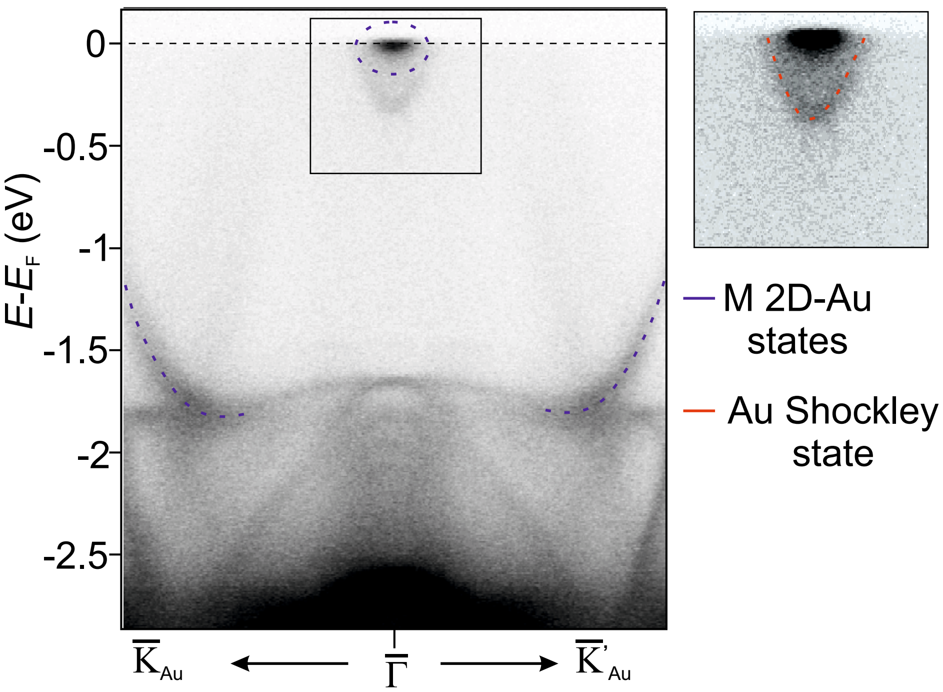

Supplementary Figure 10: **Distinction between 2D and 3D features in gold.** ARPES measurement acquired at 40 eV on a M 2D-Au on a region where also ordered 3D gold clusters were present on the surface. The M 2D-Au bands are highlighted in blue. The Au(111) Shockley state is marked with a red-dashed line in the inset of the figure.

lattice constant. A configuration very similar to what has been observed for the copper-intercalated graphene [15, 19].

Supplementary Note 7: On the nature of the feature at E_F of the M 2D-Au

To avoid possible confusion, we show in Supplementary Figure 10 an ARPES measurement acquired at 40 eV on a M 2D-Au on a region where also ordered 3D gold clusters were present on the surface. It is apparent that the feature discussed in the main text and encircled in blue in Supplementary Figure 10, is very well distinct with respect to the surface Shockley state typical of the Au(111) crystal. The Shockley state is a spin-splitted parabola centered at about -400 meV from the Fermi level [20]. That feature is clearly visible in this data set and to make it more distinguishable, we show the zoom-in of that BZ region with enhanced contrast, just next to the main figure panel. We draw a red-dashed line over the Shockley state. In such a high contrast visualization, the M 2D-Au band becomes highly prominent.

Supplementary References

[1] S. Zhou, G.-H. Gweon & A. Lanzara, "Low energy excitations in graphite: The role of dimensionality and lattice defects", *Annals of Physics* **321**, 1730 (2006).

[2] T. Ohta, A. Bostwick, J. L. McChesney, T. Seyller, K. Horn & E. Rotenberg, "Interlayer Interaction and Electronic Screening in Multilayer Graphene Investigated with Angle-Resolved Photoemission Spectroscopy", *Phys. Rev. Lett.* **98**, 206802 (2007).

[3] I. Gierz, T. Suzuki, R. T. Weitz, D. S. Lee, B. Krauss, C. Riedl, U. Starke, H. Höchst, J. H. Smet, C. R. Ast & K. Kern, "Electronic decoupling of an epitaxial graphene monolayer by gold intercalation", *Phys. Rev. B* **81**, 235408 (2010).

[4] A. L. Walter, A. Bostwick, K.-J. Jeon, F. Speck, M. Ostler, T. Seyller, L. Moreschini, Y. J. Chang, M. Polini, R. Asgari, A. H. MacDonald, K. Horn & E. Rotenberg, "Effective screening and the plasmaron bands in graphene", *Phys. Rev. B* **84**, 085410 (2011).

[5] A. Bostwick, F. Speck, T. Seyller, K. Horn, M. Polini, R. Asgari, A. H. MacDonald & E. Rotenberg, "Observation of Plasmarons in Quasi-Freestanding Doped Graphene", *Science* **328**, 999 (2010).

[6] B. Lundqvist & V. Samathiyakanit, "Single-particle spectrum of the degenerate electron gas IV. Ground state energy", *Phys. kond. Mat.* **9**, 231 (1969).

[7] E. L. Shirley, L. J. Terminello, A. Santoni & F. J. Himpsel, "Brillouin-zone-selection effects in graphite photoelectron angular distributions", *Phys. Rev. B* **51**, 13614 (1995).

[8] J. J. Paggel, W. Theis, K. Horn, C. Jung, C. Hellwig & H. Petersen, "Correlation of surface core levels and structural building blocks for the Si(111)-7×7 reconstruction through high-resolution core-level spectroscopy", *Phys. Rev. B* **50**, 18686–18689 (1994).

[9] D. Stoltz, S. E. Stoltz & L. S. O. Johansson, "A high-resolution core-level photoemission study of the Au/4H-SiC(0001)-($\sqrt{3} \times \sqrt{3}$) interface", *J. of Phys.: Cond. Matt.* **19**, 266006 (2007).

[10] J. Ristein, S. Mammadov & T. Seyller, "Origin of Doping in Quasi-Free-Standing Graphene on Silicon Carbide", *Phys. Rev. Lett.* **108**, 246104 (2012).

[11] S. Hüfner, *Photoelectron Spectroscopy*, 3rd ed., Springer Verlag (2003).

[12] C. Riedl, *Epitaxial graphene on silicon carbide surfaces: growth, characterization, doping and hydrogen intercalation*, Ph.D. thesis, Erlangen-Nürnberg University (2010).

[13] C. Riedl, C. Coletti, T. Iwasaki, A. A. Zakharov & U. Starke, "Quasi-Free-Standing Epitaxial Graphene on SiC Obtained by Hydrogen Intercalation", *Phys. Rev. Lett.* **103**, 246804 (2009).

[14] S. Forti, K. V. Emtsev, C. Coletti, A. A. Zakharov, C. Riedl & U. Starke, "Large-area homogeneous quasifree standing epitaxial graphene on SiC(0001): Electronic and structural characterization", *Phys. Rev. B* **84**, 125449 (2011).

[15] S. Forti, A. Stöhr, A. A. Zakharov, C. Coletti, K. V. Emtsev & U. Starke, "Mini-Dirac cones in the band structure of a copper intercalated epitaxial graphene superlattice", *2D Mater.* **3**, 035003 (2016).

[16] A. B. Preobrajenski, M. L. Ng, A. S. Vinogradov & N. Mårtensson, "Controlling graphene corrugation on lattice-mismatched substrates", *Phys. Rev. B* **78**, 073401 (2008).

[17] U. A. Schröder, M. Petrović, T. Gerber, A. J. Martínez-Galera, E. Grånäs, M. A. Arman, C. Herbig, J. Schnadt, M. Kralj, J. Knudsen & T. Michely, "Core level shifts of intercalated graphene", *2D Materials* **4**, 015013 (2016).

[18] B. Premlal, M. Cranney, F. Vonau, D. Aubel, D. Casterman, M. M. De Souza & L. Simon, "Surface intercalation of gold underneath a graphene monolayer on SiC(0001) studied by scanning tunneling microscopy and spectroscopy", *Applied Physics Letters* **94**, 263115 (2009).

[19] K. Yagyu, T. Tajiri, A. Kohno, K. Takahashi, H. Tochihara, H. Tomokage & T. Suzuki, "Fabrication of a single layer graphene by copper intercalation on a SiC(0001) surface", *Applied Physics Letters* **104**, 053115 (2014).

[20] D. Malterre, B. Kierren, Y. Fagot-Revurat, S. Pons, A. Tejeda, C. Didiot, H. Cercellier & A. Bendounan, "ARPES and STS investigation of Shockley states in thin metallic films and periodic nanostructures", *New Journal of Physics* **9**, 391 (2007).
